# The preventive effects of two nutraceuticals on experimentally induced acute synovitis

**DOI:** 10.1111/evj.12629

**Published:** 2016-10-13

**Authors:** E. van de Water, M. Oosterlinck, M. Dumoulin, N. M. Korthagen, P. R. van Weeren, J. van den Broek, H. Everts, F. Pille, D. A. van Doorn

**Affiliations:** ^1^ Department of Surgery and AnaesthesiologyFaculty of Veterinary MedicineGhent UniversityBelgium; ^2^ Department of Equine SciencesFaculty of Veterinary MedicineUtrecht UniversityThe Netherlands; ^3^ Department of Farm Animal HealthFaculty of Veterinary MedicineUtrecht UniversityThe Netherlands; ^4^ Equivado, Equine Nutrition ConsultancyUtrechtThe Netherlands

**Keywords:** horse, LPS, arthritis, biomarkers, pressure plate

## Abstract

**Background:**

Nutraceuticals are often used in the management of equine osteoarthritis, but scientific evidence of their efficacy is lacking.

**Objectives:**

To study the preventive effects of two new nutraceuticals after the experimental induction of synovitis in comparison with positive and negative control treatments.

**Study design:**

Blinded, controlled, randomised experiment.

**Methods:**

Twenty‐four healthy Standardbred horses were randomly allocated to supplement AT (multi‐ingredient, 28 days), supplement HP (collagen hydrolysate, 60 days), meloxicam (4 days) or placebo (60 days). Synovitis was induced in the right intercarpal joint by intra‐articular injection of 0.5 ng lipopolysaccharide (LPS) of *Escherichia coli* while treatments were continued. Blood and synovial fluid were sampled before treatment, immediately prior to LPS injection, and at 8, 24 and 48 h post‐injection. Synovial fluid samples were analysed for total nucleated cell count (TNCC), total protein (TP) and selected biomarkers (prostaglandin E_2_ [PGE_2_], interleukin‐6 [IL‐6], glycosaminoglycans [GAGs], type II collagen synthesis [CPII], matrix metalloproteinase [MMP]). Lameness was scored by visual examination and pressure plate analysis immediately prior to LPS injection, and at 8, 24 and 48 h post‐injection. Clinical examinations were performed before treatment, immediately prior to LPS injection, at 2, 4 and 6 h post‐injection, and then twice per day during the test period.

**Results:**

Before treatment and intra‐articular challenge, there were no statistically significant differences among the treatment groups for any of the parameters. After intra‐articular challenge, the placebo group showed significantly higher synovial fluid TP, TNCC and PGE_2_ compared with the meloxicam group, although the model did not induce a relevant amount of lameness. Both nutraceuticals resulted in significantly lower synovial fluid TP, TNCC and PGE_2_ compared with placebo. No statistical differences in IL‐6, GAGs, CPII or MMPs were observed among treatment groups. No adverse effects were observed.

**Main limitations:**

Despite evidence of synovitis, lameness was too mild to detect.

**Conclusions:**

The preventive administration of these nutraceuticals showed anti‐inflammatory effects in this validated synovitis model. Therefore, further studies of their clinical applicability are warranted.

## Introduction

Nutraceuticals are often used in the management of osteoarthritis, which is a common cause of chronic lameness in horses 1. However, their ‘curative’ efficacy remains controversial 2, 3 and the quality of relevant studies is generally low 4, 5. Equine in vitro studies have suggested that the combination of glucosamine and chondroitin sulphate can result in reduced cartilage degradation 6, 7, 8 and may have anti‐inflammatory effects 6, 9, 10. Although their oral bioavailability in horses is reported to be low 11, 12, an in vivo study in osteoarthritic horses showed significant clinical improvement after treatment with glucosamine and chondroitin sulphate compared with placebo treatment 13. Furthermore, methylsulphonylmethane (MSM) is a natural anti‐inflammatory agent 14 that has been found to decrease joint pain and swelling in human subjects with osteoarthritis 15, 16 and to significantly ameliorate exercise‐related oxidative and inflammatory blood changes in jumping horses 17. Finally, collagen hydrolysate showed a stimulatory effect on type II collagen biosynthesis of chondrocytes in an in vitro model with bovine chondrocytes 18. Moreover, several studies showed beneficial effects of collagen hydrolysate on joint pain associated with osteoarthritis in human subjects 19, 20. Studies of its efficacy in equine cases are lacking, but it has been found to be well absorbed and available for amino acid metabolism in horses 21.

Until now, the aforementioned nutraceuticals have only been tested using in vitro studies or in selected patients from a curative perspective. As inflammation plays a crucial role in the pathogenesis of osteoarthritis, reducing the initial inflammation can be seen as the cornerstone of preventive treatment. The efficacy of prevention can be studied using a validated synovitis model based on the intra‐articular injection of lipopolysaccharide (LPS) 22, 23. Therefore, a blinded, controlled, randomised study was designed to test the effects of two nutraceuticals using the validated model of acute synovitis described above and a comprehensive set of objective synovial and clinical variables as outcome parameters. These included quantitative locomotion analysis using a pressure plate and an array of synovial biomarkers. The hypothesis was that preventive administration of these nutraceuticals would have a beneficial effect on the degree of joint inflammation and lameness.

## Materials and methods

### Study design

The study was a blinded, controlled, randomised experiment in a block design with four treatment groups of six horses per group, all housed under the same conditions. Horses were randomly allocated to one of four dietary treatments: 1) supplement ‘AT’ (Cavalor ArtiTec [Liquid]^1^, containing glucosamine sulphate 2KCL, shark chondroitin sulphate sodium, MSM, boswellic acid dry extract 65%, *Ananasus comosus* extract 2500 GDU, l‐glutamine, feverfew dry extract PE 4:1, hyaluronic acid), administered at 45 mL twice per day for 28 days prior to articular challenge and during the 3‐day test period; 2) supplement ‘HP’ (Hydro‐P^2^, collagen hydrolysate), at 90 g once per day for 60 days prior to articular challenge and during the 3‐day test period; 3) meloxicam (Metacam^3^), at 0.6 mg/kg once per day for 4 days prior to articular challenge and during the 3‐day test period (positive control); and 4) placebo (casein), at 90 g once per day for 60 days prior to articular challenge and during the 3‐day test period (negative control).

The number of horses (n = 6) per treatment group was statistically determined using an a priori power analysis test 24 for the primary outcome variable (prostaglandin E_2_ [PGE_2_]), based on the published standard error of PGE_2_ concentration in synovial fluid in horses with synovitis 22, and a mean estimated effect of a nutraceutical treatment of 45% PGE_2_ reduction 25, at α = 0.05; β = 0.2, and an effect size of 1.68.

**Figure 1:**
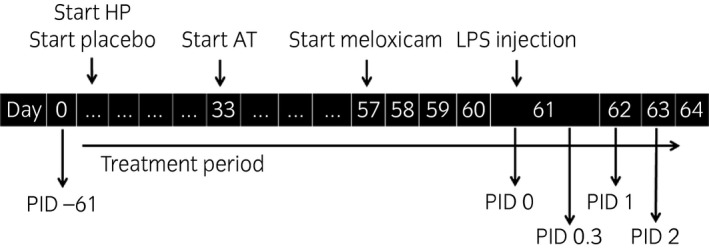
Timeline of treatments (HP, supplement HP; AT, supplement AT; LPS, lipopolysaccharide) and evaluation points, displayed by post‐injection day (PID).

Experimental procedures started 61 days before the articular challenges (post‐injection day [PID] −61), when blood and synovial fluid samples were obtained to provide baseline data before supplementation. Immediately prior to the articular challenge with LPS of *Escherichia coli* (PID 0), blood and synovial fluid were sampled and gait evaluation (visually and by pressure plate analysis) was performed to establish baseline values before the induction of arthritis. The latter procedures were repeated at 8 h (PID 0.3), 24 h (PID 1) and 48 h (PID 2) post‐injection (Fig 1). Horses were blocked in groups of four based on body weight and age. Treatments were randomly allocated within each block and blocks were entered into the study at 1‐week intervals.

### Horses

The study included 24 healthy and clinically sound female French (n = 19), Belgian (n = 4) and Dutch (n = 1) Standardbred horses (mean ± s.d. age: 9 ± 2.8 years; mean ± s.d. body weight: 495.9 ± 38.9 kg), obtained from a local breeding centre. Horses with a known history of lameness or gastrointestinal problems were excluded from the study.

### Feeding and supplementation

Horses were individually fed a standardised diet of concentrates and high‐quality hay that met their nutritional requirements 26. Horses subjected to treatment AT, meloxicam and placebo received the placebo supplement, and horses subjected to treatment HP received the HP supplement, top‐dressed over the morning concentrate ration. The morning dose AT and meloxicam were administered orally prior to the morning concentrate feed ration.

### Intra‐articular challenge

At PID 0, after baseline pressure plate analysis and arthrocentesis, synovitis was induced in the right intercarpal joint by injection of 0.5 ng LPS from *E. coli* (L5418^4^) (lot 093M4041V) in 0.8 ml sterile isotonic saline 22. Two weeks prior to the start of the study, the initial LPS solution (1 mg/ml) was aseptically diluted to a final concentration of 0.625 ng/ml. The diluted LPS solutions were stored in glass vials at 4°C.

Physical examination (respiratory rate, heart rate and rectal temperature) was performed immediately prior to LPS injection, at 2, 4 and 6 h after LPS injection, and then twice per day during the test period to control for systemic signs of endotoxaemia.

### Sampling

Synovial samples were taken at PID −61, PID 0, PID 0.3, PID 1 and PID 2. If necessary, horses were sedated with detomidine 10 *μ*g/kg and butorphanol 10 *μ*g/kg i.v. Prior to arthrocentesis, the right intercarpal joint region was clipped and aseptically prepared. With a flexed carpus, a 21 gauge, 4‐cm needle was inserted between the extensor carpi radialis and common digital extensor tendons 27. Approximately 3.5 mL of synovial fluid was withdrawn and immediately split into different sterile containers: 1 mL was collected in an EDTA‐coated tube and the remaining amount of fluid was collected in plain tubes. Samples were immediately stored at 4°C and analysed (EDTA) or processed (plain tubes) within 1 h of collection. The EDTA aliquot was analysed for cytology (total nucleated cell count [TNCC]) and total protein ([TP]). The aliquot in plain tubes was centrifuged at 600 ***g*** for 20 min, aliquoted and stored at −80°C within 2 h after collection for subsequent biomarker analysis.

Blood samples were taken from the left jugular vein with a 21 gauge, 4‐cm needle and put in silicone‐coated and EDTA‐coated vacutainers. Samples were immediately refrigerated (4°C), and subsequently transferred to the laboratory for further analysis. PID 0 samples were analysed for haematology and serum biochemistry and compared with PID −61 samples to assess treatment safety. The serum biochemistry panel included urea, creatinine, TP, albumin, α‐globulins, β‐globulins, γ‐globulins, total bilirubin, direct and indirect bilirubin, aspartate aminotransferase, γ‐glutamyltransferase (GGT), alkaline phosphatase, lactate dehydrogenase and creatine kinase. At PID 0.3, PID 1 and PID 2 only haematology was performed to assess LPS safety.

### Synovial biomarker analysis

Prostaglandins were determined by high‐performance liquid chromatography (HPLC)–tandem mass spectrometry (MS/MS) analysis following RP‐18 extraction of synovial fluid samples 28. HPLC‐MS/MS analysis was performed on a PerkinElmer LC200 HPLC system^5^ coupled to an electrospray ionisation linear ion trap quadrupole (4000 QTRAP) mass spectrometer^6^. The instrument was operated in negative MRM mode. PGE_2_ concentrations were calculated from peak areas relative to an internal standard.

Synovial fluid samples were evaluated for glycosaminoglycan (GAG) content using the 1,9‐dimethylmethyleneblue assay, adapted for use in microtitre plates 28.

Interleukin‐6 (IL‐6) was measured using the GSI Equine IL‐6 ELISA Kit^7^ for synovial fluid, in which undiluted synovial samples were found to give the best results.

CPII, a marker of type II collagen synthesis, was quantified using commercial ELISA kits^8^, as described in other studies 27, 29, 30, in accordance with the manufacturer's recommendations.

General matrix metalloproteinase (MMP) activity was measured using the fluorogenic substrate FS‐6^9^. In short, samples were diluted 20‐fold in MMP buffer (0.1 mol/l Tris, 0.1 mol/l NaCl, 10 mmol/l CaCl_2_, 0.05% [w/v] Triton X‐100, 0.1% [w/v] PEG6000, pH 7.5 and 5 *μ*mol/l FS‐6). Samples were added in triplicate to a black 384‐well microplate^10^ and the fluorescent signal was monitored continuously for 45 min at 37°C using a CLARIOstar microplate reader^11^. The slope of the resultant linear curve (relative fluorescence units/s [RFU/s]) was calculated as a measure of general MMP activity. A quantity of 5 mmol/l EDTA was used as a negative control.

### Clinical evaluation

Lameness was scored prior to arthrocentesis. At PID −61, this was performed by routine visual examination only using the American Association of Equine Practitioners (AAEP) scale, on which grade 0 represents sound ability and grade 5 indicates non weight bearing capacity, by two European College of Veterinary Surgeons diplomates, using video‐recordings 31. At PID 0, PID 0.3, PID 1 and PID 2, both video‐recordings and pressure plate measurements were used.

Pressure plate measurements were obtained using a Footscan 3D 2m‐system^12^ as described by Oosterlinck *et al*. 32. The following kinetic variables were calculated at walk and trot: 1) peak vertical force (PVF), in N/kg; 2) vertical impulse (VI), in Ns/kg; and 3) stance time (ST) in ms. For each set of five trials, all left forelimb (LF) and right forelimb (RF) measurements were averaged and subsequently PVF, VI and ST ratios between both forelimbs were calculated as: %RF = RF/(LF + RF) × 100%.

### Data analysis

Statistical analysis was performed using R (lme4 package)^13^. A linear model with random horse effects and with fixed week, time, sedation, treatment and the time–treatment interaction was used. Akaike's information criterion (AIC) was used for model reduction 33. Week and sedation were considered as block factors. If a factor was important according to the AIC, then 90% bootstrap intervals were calculated for the effect. Residuals were checked for normality using a normal probability plot. TNCC, PGE_2_, CPII and MMP data were logarithmically transformed for statistical analysis. Clinical lameness scores required a logistic regression, but with exactly the same model as described above.

## Results

### Synovial fluid analysis

**Figure 2:**
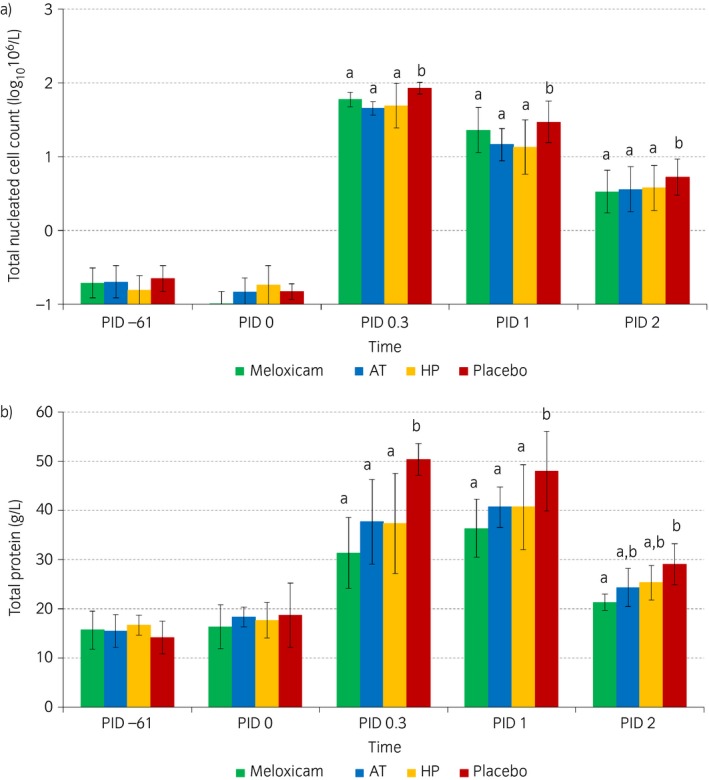
Logarithmically transformed a) mean total nucleated cell counts and b) mean total protein in synovial fluid in the different treatment groups (green: meloxicam; blue: supplement AT; yellow: supplement HP; red: placebo) over time (post‐injection days [PID] −61, 0, 0.3, 1 and 2). Within time points, outcomes in treatments with different superscripts show statistically significant differences.

The results of synovial fluid analysis are presented in Table 1. At PID −61 and PID 0, there were no statistical differences among treatment groups. After LPS injection, there was a marked increase in TNCC in all treatment groups, with a sharp peak at PID 0.3 (Fig 2a). At all time points after LPS injection, meloxicam treatment and both supplement treatments resulted in a statistically lower TNCC than placebo treatment. There were no statistically significant differences between the meloxicam group and both supplement groups.

**Table 1:** Results of synovial fluid analyses of total nucleated cell count (TNCC), total protein (TP), prostaglandin E_2_ (PGE_2_), interleukin‐6 (IL‐6), glycosaminoglycans (GAGs), matrix metalloproteinases (MMPs) and type II collagen synthesis (CPII) taken at post‐injection days (PIDs) −61, 0, 0.3, 1 and 2, in the four treatment groups (meloxicam [Melox], supplements AT and HP, and placebo [Plac])

	PID −61	PID 0	PID 0.3	PID 1	PID 2
TNCC, ×10^9^/L median (range)					
Melox	0.17 (0.12–0.41)	0.11 (0.05–0.15)	61.31 (42.93–78.15)^a^	21.89 (9.05–60.55)^a^	3.59 (1.52–8.43)^a^
AT	0.21 (0.12–0.35)	0.16 (0.10–0.29)	45.70 (31.75–61.75)^a^	15.00 (7.88–26.41)^a^	4.59 (1.21–8.16)^a^
HP	0.15 (0.09–0.31)	0.20 (0.08–0.41)	55.36 (14.52–103.40)^a^	17.90 (2.83–26.60)^a^	4.95 (1.16–6.67)^a^
Plac	0.23 (0.15–0.33)	0.15 (0.11–0.21)	80.67 (68.81–113.37)^b^	26.54 (12.78–80.35)^b^	6.89 (2.55–8.89)^b^
TP, g/L mean ± s.d.					
Melox	15.70 ± 3.88	16.33 ± 4.46	31.33 ± 7.23^a^	36.33 ± 5.85^a^	21.33 ± 1.63^a^
AT	15.50 ± 3.33	18.33 ± 1.97	37.67 ± 8.62^a^	40.67 ± 4.13^a^	24.33 ± 3.88^a,b^
HP	16.70 ± 1.97	17.67 ± 3.67	37.33 ± 10.17^a^	40.67 ± 8.64^a^	25.33 ± 3.50^a,b^
Plac	14.20 ± 3.37	18.67 ± 6.53	50.33 ± 3.20^b^	48.00 ± 8.10^b^	29.00 ± 4.15^b^
PGE_2_, pg/ml median (range)					
Melox	27.28 (9.92–143.78)	16.09 (9.02–23.10)	53.22 (19.05–127.70)^a^	33.05 (20.69–79.50)^a^	30.65 (15.45–58.01)^a^
AT	29.42 (9.95–107.28)	28.20 (8.78–99.44)	795.06 (415.43–1571.29)^b^	119.72 (96.75–308.46)^b^	130.50 (83.32–264.85)^b,c^
HP	36.04 (12.42–91.52)	30.14 (13.83–70.14)	2307.82 (234.25–12,517.59)^c^	268.88 (153.72–439.99)^b,c^	154.79 (72.01–443.62)^b,c^
Plac	36.41 (25.21–157.01)	14.98 (9.10–203.64)	3795.08 (720.28–40,960.19)^d^	304.56 (101.90–3524.93)^c^	268.31 (133.20–820.07)^c^
IL‐6, pg/ml mean ± s.d.					
Melox	3.35 ± 8.21	3.79 ± 6.10	29.69 ± 29.82	32.82 ± 44.09	15.16 ± 18.94
AT	14.56 ± 30.10	48.40 ± 106.65	126.5 ± 261.29	118.72 ± 255.30	81.37 ± 188.09
HP	6.85 ± 15.29	8.04 ± 19.70	54.92 ± 81.79	48.40 ± 91.77	25.62 ± 44.61
Plac	5.15 ± 12.62	9.40 ± 19.72	46.69 ± 60.54	29.24 ± 37.01	12.15 ± 13.93
GAGs, *μ*g/ml mean ± s.d.					
Melox	114.50 ± 42.86	108.20 ± 36.03	180.71 ± 26.99	245.61 ± 78.46	158.17 ± 49.77
AT	106.80 ± 19.78	114.22 ± 13.47	178.13 ± 24.28	241.02 ± 33.56	178.91 ± 25.94
HP	112.50 ± 29.90	115.70 ± 28.30	177.34 ± 39.77	247.95 ± 88.03	205.00 ± 71.36
Plac	125.10 ± 18.82	126.53 ± 17.10	191.41 ± 22.94	291.59 ± 78.04	230.05 ± 67.30
MMPs, RFU/s median (range)					
Melox	251.73 (157.20–451.47)	215.20 (95.67–396.80)	276.33 (157.20–568.00)	692.70 (295.40–1108.40)	418.13 (239.00–574.27)
AT	232.23 (83.20–465.40)	217.72 (152.40–317.80)	695.37 (343.67–1050.60)	929.63 (521.20–1475.87)	511.47 (270.87–703.87)
HP	244.13 (118.47–489.13)	222.77 (95.13–365.73)	493.93 (126.13–712.07)	861.80 (466.13–1300.53)	556.47 (224.67–650.33)
Plac	285.40 (115.20–440.00)	211.20 (72.80–334.20)	388.93 (33.67–967.67)	908.07 (726.73–1328.13)	592.83 (524.33–700.33)
CPII, ng/ml median (range)					
Melox	1035.91 (264.40–8681.40)	678.86 (521.07–1538.08)	1388.69 (969.47–1662.18)	1732.70 (1085.27–2535.43)	2117.65 (910.16–4948.36)
AT	1054.89 (538.99–1569.74)	1031.74 (341.15–1207.49)	1186.43 (253.31–2560.36)	3631.69 (866.49–6320.83)	4379.43 (828.20–6203.69)
HP	660.33 (373.05–1075.67)	869.76 (290.59–1094.46)	1058.04 (323.97–2289.94)	6103.99 (1032.04–8639.12)	4283.48 (1278.48–12,303.01)
Plac	436.35 (38.12–609.66)	814.47 (33.50–1883.84)	1181.77 (635.77–2874.65)	3583.98 (1117.20–12,341.05)	7653.97 (1591.68–23,051.08)

RFU/s, relative fluorescence units/s. Within time points, treatments with different superscripts show statistically significant differences.

Total protein showed a sustained increase in all treatment groups at PID 0.3 and PID 1 (Fig 2b). At PID 0.3 and PID 1, TP in the meloxicam group and both supplement groups was statistically lower than in the placebo group. At PID 2, TP in the meloxicam group only was statistically lower than in the placebo group.

**Figure 3:**
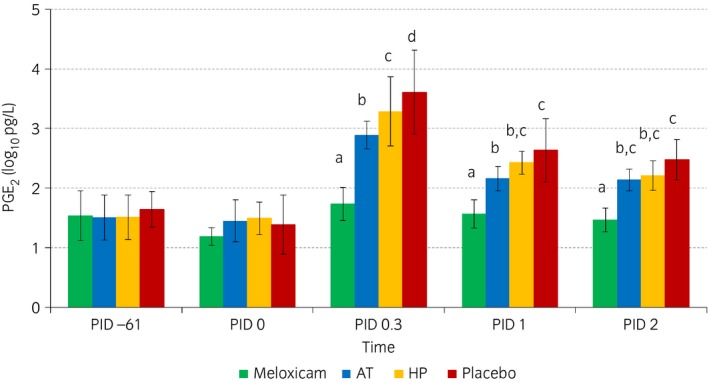
Logarithmically transformed mean prostaglandin E_2_ (PGE_2_) concentrations in synovial fluid in the different treatment groups (green: meloxicam; blue: supplement AT; yellow: supplement HP; red: placebo) over time (post‐injection days [PID] −61, 0, 0.3, 1 and 2). Within time points, outcomes in treatments with different superscripts show statistically significant differences.

Over time, the concentration of PGE_2_ showed a rise at PID 0.3 (Fig 3). IL‐6 and GAGs showed more sustained rises at PID 0.3 and PID 1, and MMPs and CPII peaked at PID 2. The PGE_2_ concentration was statistically lower in the meloxicam group than in the other treatment groups at PID 0.3. Supplement AT resulted in statistically lower PGE_2_ concentrations than supplement HP and placebo treatment, and supplement HP resulted in statistically lower PGE_2_ concentrations than placebo treatment at this time point. At PID 1, PGE_2_ was statistically lower in the meloxicam group than in all other treatment groups. Moreover, supplement AT resulted in statistically lower PGE_2_ concentrations than placebo treatment at this time point, by contrast with supplement HP. However, there was no statistically significant difference between the two supplement groups at this time point. At PID 2, PGE_2_ was statistically lower in the meloxicam group than in all other treatment groups. IL‐6, GAG, CPII and MMP concentrations showed no statistically significant differences between treatment groups at any time point.

### Lameness evaluation

Lameness was very mild (AAEP score ≤1) and visually detectable only at PID 0.3 in some horses (four of 24) and at PID 1 in one horse. Clinical lameness scores did not differ statistically between treatment groups. Even with pressure plate analysis, no statistical differences in PVF and VI left‐to‐right ratios between treatment groups could be observed at any time point. For ST, very small and inconsistent differences between treatment groups could be observed at trot after LPS injection.

### Safety of supplements and LPS injection

After supplementation (at PID 0), serum activity of GGT was increased in 21 of 24 (88%) horses (mean ± s.d.: 101.6 ± 61.9 U/L; reference range: 0–30 U/L) across all four groups, including the placebo group, and hence without any association with treatment. Other blood parameters (haematology and serum biochemistry) were within normal limits. After LPS injection, blood haematology and routine clinical examinations revealed no systemic abnormalities at any time point.

## Discussion

In vivo equine studies on nutraceuticals are usually clinical trials 13, 34, 35, which are subject to an inherent lack of standardisation. This is highlighted by the low‐quality appraisal of most nutraceutical studies, using the score list described by Pearson and Lindinger 4. To overcome the limitations of clinical studies, experimental models can be used as these provide highly standardised circumstances. Using an IL‐1β‐based experimental synovitis model, Pearson *et al*. 25 studied the effect of a nutraceutical (composed of mussel, shark cartilage, abalone and *Biota orientalis* lipid extract) on the inflammatory response in synovial fluid. In a subsequent study of another nutraceutical (a biological extract of high‐rosmarinic acid mint) 36, synovitis was induced by injection of LPS and, in addition to synovial fluid analysis, lameness was assessed as an outcome parameter, albeit subjectively. The current study is the first to show an anti‐inflammatory effect of nutraceuticals on experimentally induced synovitis using a comprehensive set of objective synovial and clinical variables, including analysis of inflammatory and cartilage biomarkers, and quantitative evaluation of locomotion, which results in an excellent quality score (>80.0) according to the classification by Pearson and Lindinger 4.

In the current study, clear synovial inflammation was observed after LPS injection, especially in the placebo group. Moreover, the registered non‐steroidal anti‐inflammatory drug meloxicam resulted in significant reductions in TNCC, TP and PGE_2_ concentrations compared with the placebo treatment, indirectly confirming the validity of the synovitis model used in this study. The resulting lameness, however, was too mild to be detectable, either visually or by pressure plate evaluation, and therefore could not be used as a parameter with which to discriminate between treatments. This contrasts with the findings of de Grauw *et al*. 22, who found a mean lameness score of 3/5 on the AAEP grading scale for the placebo treatment at 8 h post‐injection with the same LPS dose. This discrepancy is most likely attributable to differences in LPS activity between lots, but may also relate to the use of different handling procedures to create the diluted LPS solution, the use of Standardbred horses in the present study vs. Warmbloods in the study conducted by de Grauw *et al*. 22, or a combination of these factors. Breed‐dependent effects may be suspected. Pearson *et al*. 36 reported that no more than three of eight Standardbred horses showed grade 1 lameness on the AAEP scale 12 h after LPS injection in the intercarpal joint and that none of them showed any lameness at 24 h, although they used a slightly lower dose (0.3 ng LPS of *E. coli* O55:B5). Positive aspects of this issue are that horses suffered less, which is good from an equine welfare perspective, and that the subtle lameness induced resembles the clinical situation in osteoarthritis to a greater degree than the more fulminant inflammatory reaction described by de Grauw *et al*. 22. Nevertheless, a slightly higher degree of lameness would have permitted quantitative gait analysis.

The less pronounced lameness in the present study in comparison with that reported by de Grauw *et al*. 22 was mirrored by a lower peak TNCC and lower maximal CPII concentration than described in the earlier paper and reflects a slightly lower degree of inflammation. However, the effectiveness of the experimental model is illustrated by the statistically relevant increases in cell counts and inflammatory biomarkers in the placebo group and the statistically significant differences between the meloxicam and placebo treatments for TNCC and TP. The maximal concentrations of PGE_2_ and GAG were similar to those described by de Grauw *et al*. 22, but by contrast with the study conducted by de Grauw *et al*. 23, the present study did not reveal a statistically significant difference in GAG content between the meloxicam and placebo treatments.

The statistically lower TNCC, TP and PGE_2_ concentrations after LPS injection in both supplement groups compared with the placebo treatment group prove that both supplements have anti‐inflammatory effects. As inflammation plays an important role in the pathogenesis of osteoarthritis 37, 38, it is plausible that these supplements may be of benefit during the developmental stage of osteoarthritis. Unfortunately, the acute, temporary synovitis model does not allow for the drawing of conclusions about possible long‐term effects.

Apart from an increase in serum GGT activity, no changes in blood haematology or serum biochemistry were observed after supplementation. Increased GGT activity was observed in all treatment groups, including the placebo group, which suggests it was not associated with the supplementation; therefore, the present authors conclude that the periods of supplementation with the products described here (28 days for AT and 60 days for HP) can be considered safe. The reason for the increased GGT activity in all groups remains unclear. The horses did not show any clinical symptoms of liver disease or any other abnormalities. There was no dietary change during the study.

The limitations of the current study are that the clinical evaluation of horses’ locomotion was based on video‐recordings, and that pressure plate analysis was not performed prior to articular challenge. There are inherent limitations in the evaluation of locomotion using video‐recordings compared with a full clinical examination, but the use of video‐recordings avoided observer bias at individual time points. Moreover, pressure plate analysis confirmed the absence of relevant asymmetry in horses’ locomotion. At PID −61, measurements were performed at a breeding farm at which a large herd of horses was available, whereas the induction of synovitis and pressure plate analysis were performed at the university hospital. Pressure plate equipment was not available at the breeding farm. However, the aim of the study was not to compare locomotion before and after supplementation with nutraceuticals, but to evaluate the effects of the preventive administration of nutraceuticals on the acute synovitis induced with LPS.

## Conclusions

The nutraceuticals investigated in this study ameliorated experimental joint inflammation in a validated synovitis model. Therefore, the clinical benefits of these products in patients with various degrees of joint pathology should be evaluated.

## Authors’ declaration of interests

D.A. van Doorn was previously employed by Nutriquine NV and hired in his role as an equine nutrition consultant by the funders of this project (Nutriquine NV, Drongen, Belgium and Sonac BV, Son, the Netherlands) to co‐ordinate the execution of the project. He does not receive any royalties relating to the products tested in this trial. The funding companies did not participate in analysis or the decision to publish. All authors declare they had full autonomy and independency in research and publishing.

## Ethical animal research

The study was approved by the ethical committee of the Faculty of Veterinary Medicine of Ghent University (no. 2013/165).

## Sources of funding

This study was funded by Nutriquine NV (Drongen, Belgium) and Sonac BV (Son, the Netherlands).

## Authorship

E. van de Water, M. Oosterlinck, F. Pille, M. Dumoulin and D.A. van Doorn contributed to the study design and execution, data analysis and interpretation, and the preparation of the manuscript. N.M. Korthagen, P.R. van Weeren, J. van den Broek and H. Everts contributed to the study design, data analysis and interpretation, and the preparation of the manuscript. All authors approved the final manuscript.

## Supporting information


**Summary in Chinese.**

## Manufacturers' addresses

1 Nutriquine NV, Drongen, Belgium.

2 Sonac BV, Son, the Netherlands.

3 Boehringer Ingelheim Vetmedica, Ingelheim/Rhein, Germany.

4 Sigma‐Aldrich Corp., St Louis, Missouri, USA.

5 PerkinElmer, Inc., Waltham, Massachusetts, USA.

6 Applied Biosystems International, Inc., Bleiswijk, the Netherlands.

7 Genorise Scientific, Inc., Glen Mills, Philadelphia, USA.

8 IBEX Pharmaceuticals, Inc., Quebec, Canada.

9 Brachem AG, Bubendorf, Germany.

10 Greiner Bio‐One GmbH, Frickenhausen, Germany.

11 BMG Labtech, Ortenberg, Germany.

12 RSscan International NV, Paal, Belgium.

13 R Foundation for Statistical Computing, Vienna, Austria.
