# Supplementary material for: The preventive effects of two nutraceuticals on experimentally induced acute synovitis
Source: Equine Vet J. 2016 Oct 13;49(4):532–8. doi: 10.1111/evj.12629 (PMC5484312; doi:10.1111/evj.12629)
Supplement: Supplementary file 1 — Summary in Chinese. [file EVJ-49-532-s001.docx]

**EVJ-GA-16-038.R1**

**2种添加剂保健品对实验诱导的急性滑膜炎的预防作用**

E. Van de Water^1*^, M. Oosterlinck^1^, M. Dumoulin^1^, N.M. Korthagen^2^, P.R., van Weeren^2^, J. van den Broek^3^, H. Everts^3^, F. Pille^1,δ^, D.A. van Doorn^2,3,4,δ^

**关键词：**马； LPS；关节炎；生物标志物；压板

**摘要**

**背景：添加剂**保健品常被用来辅助处理马匹骨关节炎，但是缺乏其功效的科学证据。

**目的：**与阳性和阴性对照处理相比，研究两种新的添加剂保健品对实验诱导滑膜炎的预防效果。

**研究设计：**双盲、对照，随机试验

**方法：**24匹健康标准竞赛马随机分组为补充添加剂AT（多配料，28天）、补充添加剂HP（水解胶原蛋白，60天）、美洛昔康（4天）或安慰剂（60天）。通过在关节腔内注射大肠杆菌脂多糖0.5ng诱导右腕关节内滑膜炎，同时继续治疗。血液和滑膜液样品分别在处理前、LPS注射前立即、注射后8、24、和48h。滑膜液样品分析：总有核细胞计数（TNCC）、总蛋白（TP）和选定的生物标志物（PGE_2_、IL-6、GAGs，CPII、MMP）。跛行在LPS注射前立即和注射后8、24和48h，通过目测观察和压力板分析打分。临床检查在处理前、LPS注射前立即、注射后2、4、6h，之后每日两次至试验结束。

**结果：在**处理及关节内注射前，不同处理组间无任何参数有统计学差异。关节内注射后，与美洛昔康组相比，安慰剂组滑膜液TP、TNCC和PGE_2_统计学显示更高，但该模型并未引起一定程度的跛行。与安慰剂相比，两种保健品组统计学上滑膜液TP、TNCC和PGE_2_降低_。_不同处理组间未观察到IL-6、GAGs、CP-II和MMPs的统计学差异。没有观察到不良反应。

**主要局限：**尽管滑膜炎有实验室证据，但跛行太轻微不能检测。

**结论：**这些保健品的预防性给药，在此验证了的滑膜炎模型中显示了抗炎作用，因此进一步的临床应用研究很有必要。
